# Surgical laser positioning system assists removal of a cervical migratory foreign object

**DOI:** 10.1016/j.bjorl.2021.03.013

**Published:** 2021-04-27

**Authors:** Jianghong Wu, Panyu Zhou, Demeng Xia, Lei Li, Sheng Wang, Shuogui Xu

**Affiliations:** a Second Military Medical University (Naval Medical University), Changhai Hospital, Department of Orthopedics, Shanghai, China; b Second Military Medical University (Naval Medical University), Changhai Hospital, Department of Emergency, Shanghai, China

## Introduction

The neck, which is often exposed to the environment, is a common site of foreign object injuries.^1^ Many important structures are located in this region, including the thyroid, trachea, esophagus, major blood vessels and nerves. When cervical trauma occurs due to the insertion of foreign objects, mechanical injury and inflammatory reactions may lead to bleeding, perforation, asphyxia, and other severe complications. Hence, complete removal of foreign objects are of vital importance for patients with cervical foreign bodies. However, due to the existence of numerous cavities, the traction force of cervical muscles, respiratory movement, the surgical procedure itself, and other factors, foreign objects have a tendency to migrate through cervical tissues. They can even migrate long distances along blood vessels and nerves. Their position can change before or even during surgery. Inaccurate positioning of foreign objects poses great difficulties for surgery.^2^, ^3^ In this article, by reporting a case of a traumatic cervical foreign object, we introduce a novel laser positioning navigation technique, called the “surgical approach visualization and navigation” (SAVN) system. It can assist positioning accurately and allow visualization during surgery.

## Case report

### Clinical history

**Figure 1:**
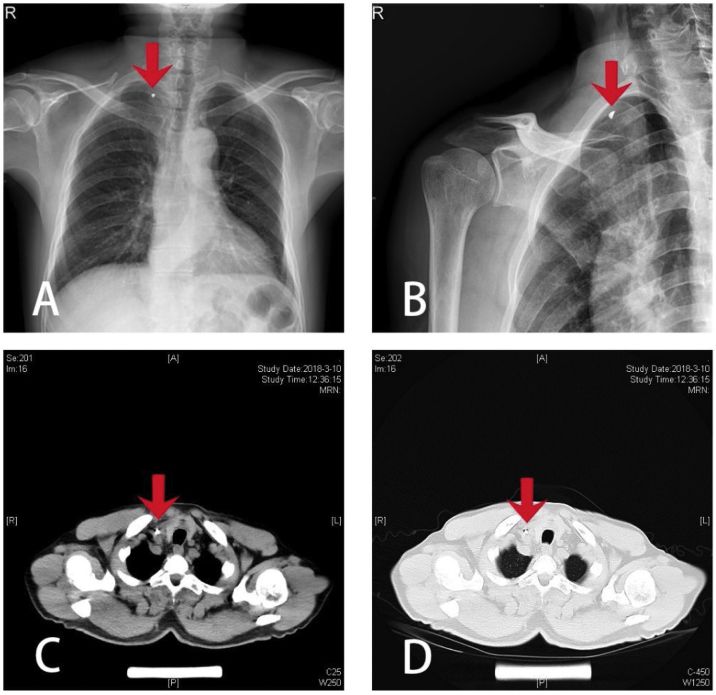
Preoperative imaging: (A) frontal chest radiograph; (B) tangential clavicular joint radiograph; (C) chest CT plain scan (mediastinal window); (D) chest CT plain scan (pulmonary window); (red arrow pointing at the foreign object).

A 65-year-old male patient was performing a striking motion with an iron hammer when debris from the iron fell off the hammer, rebounded on the wall behind him, flew from above the head, descended in a right downward direction and penetrated his right cervical root, leaving an 0.8 cm long irregular wound at the posterior margin of clavicular end of the sternocleidomastoid muscle, with local redness and swelling but no obvious bleeding. The patient's vital signs at the time of injury were stable. The patient went to a local hospital on the third day after injury. Imaging examinations showed that a metal foreign object was located at the middle lower part of the sternocleidomastoid muscle, slightly to the interior. The location of the foreign object was deep and was highly likely to be clinging to the carotid artery (Fig. 1). The local hospital was not capable of carrying out the surgery, so the patient was transferred to our hospital. After preoperative routine tests were done, we prepared the patient for a SAVN system-guided surgical removal of the cervical foreign object under general anesthesia.

### Surgical procedures

**Figure 2:**
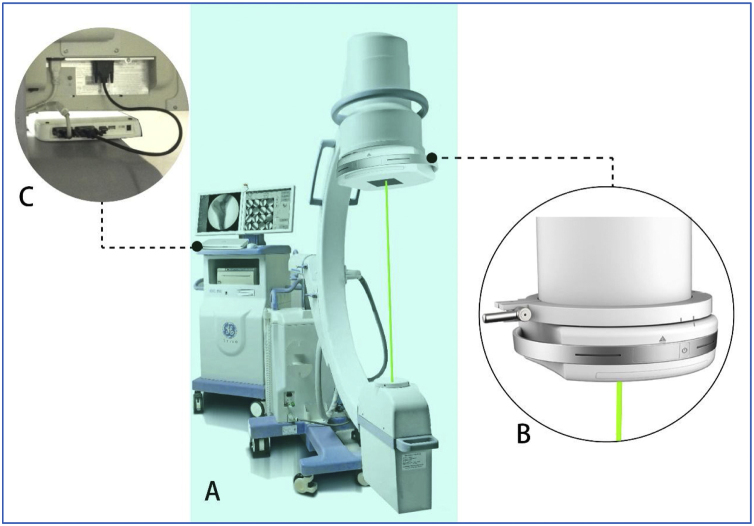
Components of the SAVN system: (A) the navigator is built on a C-arm machine; (B) laser driving device; (C) data processing module.

**Figure 3:**
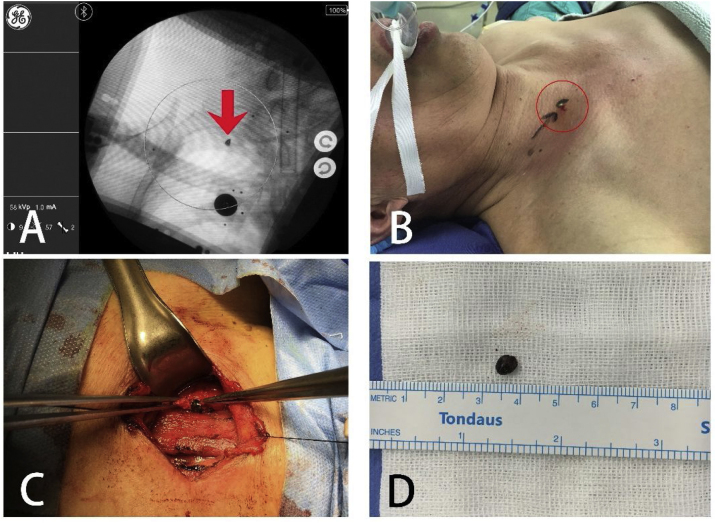
Surgical process: (A) foreign object is indicated by SAVN system; (B) foreign body is indicated by laser spot on body surface, which is a green shining spot emphasized by a red circle; (C) foreign body taken out from the carotid sheath; (D) the metal foreign object.

The patient was placed in a supine position under general anesthesia. Normal sterilization and drape procedures were subsequently carried out according to standard protocols. The SAVN system was installed on the C-arm machine and debugged (Fig. 2A). An image was taken when the C-arm was placed in the right position, showing the clear presence of the metal object located at the middle lower part of the sternocleidomastoid muscle, slightly to the interior (Fig. 3A). We selected the shown location of the object on the C-arm screen by clicking the mouse, and the laser driver device (Fig. 2B) automatically adjusted the position and angle of the built-in laser emitter according to the position information transmitted by the data processing module (Fig. 2C), and simulated X-ray emission by laser, which penetrates the foreign object with visible laser. The light spot on the body surface indicated the perpendicular projection of the foreign object and was about 0.5-cm above the wound where the metal was inserted (Fig. 3B). We then made a 3 cm long transverse incision using cis-cleavage lines at the laser positioned spot. The tissues were then separated layer by layer. We explored from the positioned spot into deeper layers, until reaching the carotid sheath. The wall of the sheath was intact, and no blood clot was observed. Then, we reran the laser positioning procedure, which still showed that the foreign object was located in the same position. Therefore, we presumed that the foreign object may have entered the carotid sheath from elsewhere. We opened up the carotid sheath, carefully separated the internal jugular vein from the common carotid artery, explored carefully according to laser positioning information, eventually found the foreign object at the lateral wall of the carotid sheath and removed it (Fig. 3C). No vascular damage was caused during the procedure, and the foreign object, a 0.5 × 0.6 × 0.4 cm flat iron fragment, was removed intact (Fig. 3D). The fragment matched the broken portion of the hammer.

In the preoperative discussion, judging by the oblique right downward trajectory of the foreign object, we considered that the metal object was likely to slide a certain distance down the soft tissue after penetrating the root of the neck, and could be lodged in the wound tract below the surface of the wound. However, intraoperative laser positioning showed that the foreign object was located about 0.5 cm above the wound. Additionally, we found that the wall of the carotid sheath under the positioned spot was intact. We thus speculated that the foreign object did not enter the wall of the vascular sheath at the positioned spot. Instead, after shooting into the cervical root, it probably entered the wall of the vascular sheath from beneath its surface entry. Then, the wave-like movement of the vessel wall caused by arterial pulsation moved the object from the proximal end to the distal end, which probably caused the foreign object to move upward along the vascular sheath. Therefore, its location had changed compared to the point of initial identification.

To verify our speculation and to explore whether there was blood vessel damage, we extended the original incision to 6 cm, and further explored the clavicular joint along the trajectory of the foreign object. An approximately 0.5 cm breach was seen on the wall of the carotid artery sheath behind the clavicular joint, while a few visible pieces of dark red blood clots were attached around the breach. After careful exploration, we found no residue of foreign objects and no other vascular damage. It could be inferred that the foreign object had entered the wall of the carotid artery from the posterior part of the clavicular joint, and then it moved upwards to the intraoperative laser positioned location. Fluoroscopy was rerun to make sure there was no more foreign object residue. After routine irrigation, the incision was sutured in layers. Fluoroscopy was run 6 times and there was 20 mL of blood loss during the whole surgery. No blood transfusion was required.

## Discussion

The key to the removal of foreign objects is accurate positioning. Accurate positioning and minimizing procedure-related migration and tissue damage are especially important when the location of the foreign object is deep, or when it is adjacent to vital organs and has the risk of migration. Clinical histories and preoperational imaging can provide important data for the location of foreign objects, but in the neck or other body parts where soft tissues are abundant, foreign objects are usually small, deeply located, and have a high risk of migration.^4^ Therefore, preoperative imaging can only be used as a reference, which is of limited help for the operating surgeon. Hence, in order to ensure successful operation, intraoperative positioning is required.

Here, we introduce a novel intraoperative positioning system. This system is a new technology that uses a “X-ray trajectory visualization during target imaging” modality. On the basis of X-ray fluoroscopy with a C-arm machine, SAVN simulates the X-ray positioning of an object by means of a visual laser assisted by computer in order to realize accurate positioning.^5^, ^6^ For specific application to migratory foreign object soft tissue injuries, a laser positioning and navigation system has some advantages. First, the laser positioning process is carried out without any direct contact with the operation area. This not only eliminates the mechanical interference of the positioning process to the tissues, but also makes the operator's objectives clearer, which can effectively reduce the tissue traction caused by the operation and prevent the foreign object from further migrating. Second, if a foreign object migrates during the surgery, the SAVN system can quickly reposition the object without the need to readjust the system, thus reducing the secondary damage caused by foreign object migration. Third, the minimum spot diameter of the laser beam can be 0.2 mm, which meets the high precision requirement for the positioning small foreign objects. Lastly, at the beginning of the surgery, the shape of the foreign object can be outlined with a marker pen on the body surface to guide the selection of incision, so the surgery can be minimally invasive.

In this case, a foreign object migrated into the vascular nerve sheath after the initial injury. It entered the carotid sheath from the back of the sternoclavicular joint and moved upward. Fortunately, under the guidance of the SAVN system, the foreign object was successfully removed without any neurovascular injury. Up to now, the literature reports migratory cervical foreign objects are mostly focused migratory esophageal foreign objects after fistula formation, or long distance migration of foreign objects inside blood vessels. Cases of migration inside the vascular nerve sheath are rarely reported. What we have learned for this case was as follows. First, the neck is rich in soft tissues and has a complex anatomy, with major blood vessels and nerves distributed in this area. Surgeries in this area call for proficient mastery of the underlying anatomy. Close attention should be paid to migration-related blood vessel damage, to avoid hematoma or massive bleeding. Second the timing of surgery is critical for minimizing damage. Surgery should be carried out as early as possible in order to reduce the risk of migration-related injuries. Third, preoperative imaging, including X-Ray fluoroscopy, ultrasound, CT scan, and MRI, are all important references for the positioning a foreign object and deciding the optimal operational approach. Preoperative imaging data can provide important information about the material characteristics of the foreign objects, its anatomical relationship with the surrounding structures, and the number of foreign objects present. Moreover, preoperative imaging is also significantly helpful for detecting the occurrence of migration and the presence of secondary injuries.

## Conclusion

In this case, we found the cervical foreign object migrated in the vascular sheath, making it difficult to remove. With the assistance of the laser positioning system, the rapid and accurate positioning and removal of foreign objects can be achieved, which is useful, and worth being considered.

## Conflicts of interest

The authors declare no conflicts of interest.
